#  Lipid network and moiety analysis for revealing enzymatic dysregulation and mechanistic alterations from lipidomics data

**DOI:** 10.1093/bib/bbac572

**Published:** 2023-01-02

**Authors:** Tim D Rose, Nikolai Köhler, Lisa Falk, Lucie Klischat, Olga E Lazareva, Josch K Pauling

**Affiliations:** LipiTUM, Chair of Experimental Bioinformatics, TUM School of Life Sciences, Technical University of Munich, 85354 Freising, Germany; LipiTUM, Chair of Experimental Bioinformatics, TUM School of Life Sciences, Technical University of Munich, 85354 Freising, Germany; LipiTUM, Chair of Experimental Bioinformatics, TUM School of Life Sciences, Technical University of Munich, 85354 Freising, Germany; LipiTUM, Chair of Experimental Bioinformatics, TUM School of Life Sciences, Technical University of Munich, 85354 Freising, Germany; Chair of Experimental Bioinformatics, TUM School of Life Sciences, Technical University of Munich, 85354 Freising, Germany; Division of Computational Genomics and Systems Genetics, German Cancer Research Center (DKFZ), 69120 Heidelberg, Germany; Junior Clinical Cooperation Unit Multiparametric methods for early detection of prostate cancer, German Cancer Research Center (DKFZ), Heidelberg, Germany; European Molecular Biology Laboratory, Genome Biology Unit, 69117 Heidelberg, Germany; LipiTUM, Chair of Experimental Bioinformatics, TUM School of Life Sciences, Technical University of Munich, 85354 Freising, Germany

**Keywords:** network enrichment, lipid metabolic networks, lipidomics, disease mechanisms

## Abstract

Lipidomics is of growing importance for clinical and biomedical research due to many associations between lipid metabolism and diseases. The discovery of these associations is facilitated by improved lipid identification and quantification. Sophisticated computational methods are advantageous for interpreting such large-scale data for understanding metabolic processes and their underlying (patho)mechanisms. To generate hypothesis about these mechanisms, the combination of metabolic networks and graph algorithms is a powerful option to pinpoint molecular disease drivers and their interactions. Here we present lipid network explorer (LINEX$^2$), a lipid network analysis framework that fuels biological interpretation of alterations in lipid compositions. By integrating lipid-metabolic reactions from public databases, we generate dataset-specific lipid interaction networks. To aid interpretation of these networks, we present an enrichment graph algorithm that infers changes in enzymatic activity in the context of their multispecificity from lipidomics data. Our inference method successfully recovered the MBOAT7 enzyme from knock-out data. Furthermore, we mechanistically interpret lipidomic alterations of adipocytes in obesity by leveraging network enrichment and lipid moieties. We address the general lack of lipidomics data mining options to elucidate potential disease mechanisms and make lipidomics more clinically relevant.

## Graphical Abstract

**Graphical Abstract:**
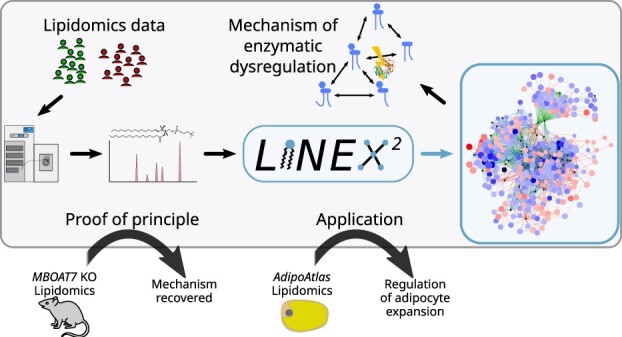

Lipid network explorer (LINEX$^{2}$) is a framework to visualize and analyze quantitative lipidomics data. The included algorithms offer new perspectives on the lipidome and can propose potential mechanisms of dysregulation.

## Introduction

Lipids play a fundamental role in cells across all domains of life. They are not only crucial for the long-term storage of energy but can also influence the activity and occurrence of membrane proteins [1], as well as signaling and inflammatory processes [2, 3]. Therefore, diseases are also influenced by lipids. This is known not only for liver and metabolic diseases [4, 5] but also, e.g. various cancers [6–9]. Despite their essential role in many biological processes, excessive accumulation of lipids, especially in non-adipose tissues can lead to lipotoxicity [10, 11]. Hence, to fully understand diseases on the molecular level, changes in the lipidome have to be characterized and their regulation understood.

Nowadays, an increasing part of the lipidome can be identified and quantified using mass spectrometry (MS). Lipidomics, is becoming more relevant for clinical applications [12], potential biomarkers have been discussed [13–15] and disease stratifications based on lipidomics proposed [16, 17]. To gain more insights into disease mechanisms, it is necessary to propose functional interpretations of lipid changes and links to other omics layers. Due to the complexity of both acquired lipidomics data as well as the regulatory mechanisms behind lipid metabolism, dedicated computational tools are of great importance for unraveling these associations.

Such interactions can be studied through biological networks. On the metabolic level, these networks describe reactions between metabolites that are catalyzed by enzymes. When considering lipid metabolic networks an additional constraint is the inherent complexity of the lipidome and its chemical reactions. One lipid enzyme usually catalyzes a reaction for a group of lipids that, e.g. belong to one lipid class but differ in their fatty acyl composition. This is reffered to as multispecifity [18]. The combinatorial complexity makes generating lipidome scale metabolic networks for an organism inefficient but instead requires data-specific networks [19–21].

Metabolic networks are commonly studied with dynamic modeling or constraint based modeling. These techniques allow predictions of the system dynamics, for example the distribution of energy resources. Parameterization of such models requires large amounts of data covering the entire molecular state [22]. Especially metabolic fluxes and well-characterized enzyme kinetics are important, which are often not available in a clinical setting.

Another way to analyze biological networks is through network enrichment. By comparing two experimental conditions, the goal is to find highly connected molecular subnetworks that are enriched with significant features. The rationale behind this approach is to propose a mechanistic hypothesis for observed dysregulations. Many algorithms have been developed over the years [23–27], mainly with a focus on protein–protein interaction (PPI) or gene-regulatory networks. A dedicated method for metabolomics data is included in the MetExplore software [28]. Their MetaboRank [29] algorithm is a network fingerprint recommendation method. For lipid networks, the BioPAN software generates lipid networks and identifies active reaction chains [20, 21]. However, operates only on the lipid sum species level and identifies only linear reaction chains. The shiny GATOM method [30] performs a network enrichment for lipids based on the Rhea reaction database. Additionally, the software is able to include gene expression data for enzymes. We previously developed the lipid network explorer (LINEX) [19], which addresses this. It combines lipid class and fatty acid metabolism to provide comprehensive networks for computational analysis and lipidomics data interpretation. Using the LINEX framework we showed that new insights into lipidome-wide data can be generated using lipid networks and that central alterations are often metabolically highly related [19]. A limitation is that lipid class reactions beyond the default have to be entered by users, which requires detailed knowledge about lipid metabolism. In contrast to *de-novo* network enrichment, pathway enrichment identifies significantly altered categorized pathways. For metabolites, this can be performed with the KEGG [31] or Reactome database [32]. A recent lipid-specific method is the Lipid Ontology web service (LION/web), which performs an ontology-based enrichment incorporating biological and chemical properties of lipids [33]. So far, no method is available, that puts the multispecifity of lipid enzymes into the center of interpreting lipidomic changes.

Here we present LINEX$^2$, a redesigned and extended framework, which addresses the shortcomings of lipid-network based methods. Lipid reactions are based on database information. This provides links to other omics disciplines. Furthermore, we developed a lipid-network enrichment algorithm, that incorporates multispecific enzyme links. The method enables the generation of mechanistic hypothesis from lipidomics data. We successfully applied our method to lipidomics data of a knock-out study and reveal potential dysregulations of the lipid metabolism in the adipose tissue of obese humans. This can help to better translate lipidomics into clinical application [34, 35] and improve our understanding of the role of lipid metabolism in disease mechanisms.

## Materials & Methods

### Database parsing & curation

We obtained lipid-related reactions from the Rhea [36] and Reactome [32] databases. From Rhea, all reactions involving lipids were parsed (based on ChEBI ontology, a subclass of CHEBI:18059). All reactions included in the category ‘Metabolism of Lipids’ for all available organisms (e.g. R-HSA-556833 for *Homo sapiens*) were parsed from Reactome.

After parsing, all lipids and reactions were manually curated. Lipids were annotated and assigned to classes according to an updated version of lipid nomenclature from Pauling *et al*. [37] with 107 lipid classes (Supplementary Table S1). Lipids are commonly composed of a headgroup, a backbone and a set of attached fatty acids. From the databases, we extracted reactions showing conversions between common lipid classes, which are usually based on changes in one of these three attributes of lipids. We classified these lipid class reactions with at least one annotated lipid available into different categories: headgroup modification (e.g. PS $\leftrightarrow $ PE), headgroup addition/removal (e.g. DG $\leftrightarrow $ PA), fatty acid addition/removal (e.g. LPC $\leftrightarrow $ PC), lipid merging (e.g. PA + PG $\leftrightarrow $ CL) (see next section and Supplementary Figure S1 for more detailed descriptions). Fatty acid reactions on complex lipids are heuristics and can be manually added or banned by the user. Default available reactions are fatty acid elongation (increasing the chain length by 2), fatty acid desaturation (adding one double bond) and hydroxylation/oxidation (adding one hydroxylation/oxidation to a fatty acid).

### Network extension to species level

Curated class reactions from databases are used to infer lipid species networks. To properly evaluate the reactions, molecular lipid species are required. This means that for each lipid the attached fatty acid must be available. Therefore, all lipid species, which are only available as sum species, are converted into a set of possible molecular species. As an example, a PC(40:2) has to be converted into possible molecular species such as PC(20:0_20:2) or PC(22:2_18:0). For this, possible common (class-specific) fatty acids can be added by the user. Only if at least one molecular species can be generated that has the same sum formula as the original sum species, it is considered for the network extension.

Extension of lipid class metabolic networks to lipid species networks can be divided into two steps: extension of the class metabolism and fatty acid metabolism. A detailed explanation can be found in the Supplementary Methods.

### Network enrichment

We developed a novel network enrichment algorithm for lipid networks. The methodology involves 1) building a reaction network from a LINEX$^2$ network and calculation of substrate-product changes per reaction. 2) Utilization of a local search algorithm to find the heaviest connected subgraph (i.e. the subgraph with the largest average substrate-product change) and 3) an empirical *P*-value estimation. All steps are described below.

#### Reaction network building

To convert the lipid network to a reaction network, we generate a unique reaction identifier for each reaction (edge) in the network extension. This is especially important for reactions with more than one substrate and product, with multiple edges corresponding to one lipid species reaction. In the next step, all lipid species reactions are converted to a new network representation with reactions as nodes. Edges between two reaction nodes are drawn, if the reaction belongs to the same lipid class reaction or at least one lipid species can be found in both reactions.

**Substrate-product change calculation** Substrate-product changes are calculated using the lipidomics data matrix $L = \mathbb{R}^{l \times n}$ consisting of $l$ lipids and $n$ samples. Samples are assigned either to the disease condition $D=\{d_1..d_x\}$ or to the control condition $C=\{c_1..c_z\}$. The score is calculated independently for each reaction $r_i$ for all reactions $R$. A reaction $r_i$ is a subsets of lipids that participate in the reaction as substrates $S(r_i)$ or products $P(r_i)$. The absolute substrate product difference $\Gamma ^a$ for reaction $r_i$ for of the disease samples $D$ is calculated as:

$$ \begin{align*}& \Gamma_{r_i}^{a,D}=\frac{\sum_{d\in D}\left(\frac{1}{|P(r_i)|}\sum_{p\in P(r_i)} L_{p, d}-\frac{1}{|S(r_i)|}\sum_{s\in S(r_i)} L_{s, d}\right)}{|D|}. \end{align*} $$

Similarly, the relative substrate–product difference $\Gamma ^r$:

$$ \begin{align*}& \Gamma_{r_i}^{r,D}=\frac{\sum_{d\in D}\left((\prod_{p\in P(r_i)} L_{p, d})^{\frac{1}{|P(r_i)|}} / (\prod_{s\in S(r_i)} L_{s, d})^{\frac{1}{|S(r_i)|}}\right)}{|D|}. \end{align*} $$

Within the calculation, the mean or root is used to correct for bias towards reactions with multiple products or substrates. The final score for each reaction node in the network is then calculated as follows:

$$ \begin{align*}& \text{Score}(r_i)=\frac{\left|\Gamma_{r_i}^D-\Gamma_{r_ii}^C\right|}{\Gamma_{r_i}^C}. \end{align*} $$

As previously explained, reactions of the fatty acid metabolism or ether lipid conversions are heuristic, to improve network connectivity. They do not occur directly on the lipid level. For that reason, they are also considered in the network enrichment but penalized (default = –1) to favor the selection of the non-heuristic reactions.

#### Local search and simulated annealing

Local search optimization investigates the search space by applying local changes to candidate solutions, such that the objective function value is increasing. The changes are applied until no more local improvements can be made. To avoid stagnation in a local maximum, the simulated annealing procedure [38] allows non-optimal solutions and thus increases the exploration space. The probability of accepting a suboptimal solution depends on the temperature parameter $T$, which decreases over time at rate $\alpha $:

$$ \begin{align*}& T = T_0 \cdot \alpha^n \end{align*} $$

where $T_0$ is the initial temperature, $\alpha $ is the rate of decrease and $n$ is the iteration number. If no more local improvements are possible, a random solution is accepted under the following condition:

$$ \begin{align*}& e^{\frac{o_{n-1}-o_n}{-T}}>\text{uniform}(0,1) \end{align*} $$

where $o_{n-1}$ and $o_n$ are objective function scores at iterations n-1 and n correspondingly.

We employ local search on the reaction network $G = (V,E)$. Starting from a (random) set of connected starting nodes, also called seed, the local search can perform three actions for improvement in the objective function scores: node addition, node deletion and node substitution. A minimum and maximum size for the subnetwork have to be entered as parameters, preventing the algorithm from selecting too small or big solutions. The action that allows improving the current value of the objective function is accepted, and thus a candidate solution is modified at each iteration. The algorithm terminates when a) no further improvements are possible, b) the simulated annealing condition is not satisfied or c) the number of maximum iterations is reached. The best-identified subnetwork is returned. The objective function score of a reaction subnetwork $G^*=(V^*, E^*)$ is computed as follows:

$$ \begin{align*}& o=\frac{\sum_{v_i\in V^*}\text{Score}(v_i)}{|V^*|\times(p\times|\text{CR}(V^*)|)} \end{align*} $$

with a user defined penalty $p$ for the number of different lipid class reactions in the subnetwork and $CR(V^*)$, the set of different lipid class reactions in the set nodes $V^*$. If the reaction network consists of unconnected components, the local search is run for each component independently and a subgraph for each component is returned.

#### Subnetwork *p*-value

The network enrichment algorithm results in a subnetwork with a score for each run. To indicate if this subnetwork/score provides a significant insight compared to an equally sized random set of reactions, we compute an empirical *P*-value. For that, we sample reactions in the range of the minimum and maximum subnetwork size. These reactions are not connected, as in the subnetwork of the enrichment. This creates a distribution of scores. The distribution is then used to estimate a *P*-value for the solution found by the enrichment. The number of samples can be decided by the user, with more samples giving a better estimate of the distribution at increased runtime. The rationale behind sampling unconnected solutions is to estimate how much the connected (mechanistic) subnetwork scores compared to unconnected (non-mechanistic) solutions.

### Reaction ratio plots

Visualizations of reaction ratios were performed for each lipid class reaction individually. Reaction ratios per sample are computed in the same as for the substrate–product change calculation, without averaging over all individuals:

$$ \begin{align*}& \frac{\left(\prod_{p\in r_i} p_{n}\right)^{\frac{1}{|p|}}}{\left(\prod_{s\in r_i} s_{n}\right)^{\frac{1}{|s|}}} \end{align*} $$

All ratios per experimental condition are compiled into a list and the density for each considered experimental condition is plotted.

### Lipid moiety analysis

The (combined) abundance of lipid features was implemented inspired by the glycan substructure method by Bao *et al*. [39]. We used the same vectorization and weighting as the authors, but with lipid substructures as features. These were: headgroup, backbone, independent fatty acyls, sum length of fatty acyls, sum double bonds of fatty acyls and fatty acyl hydroxylations. The features were weighted independently or in combination of pairs by occurrence in each lipid per sample. To find the most discriminative feature combinations, we train a regression model with sample groups as target variables and extract its coefficients. A summary of the workflow can be found in Supplementary Figure S2.

### Analyzed data sets

The lipidomics data for MOBAT7 WT and knockout mice were taken from Thangapandi *et al*. [40]. The data contain 16 knockout samples and 14 WT samples with 244 lipids from 18 lipid classes. No further processing was done and the data were analyzed as provided by the authors. Data for the Adipo Atlas were used as provided in the supplement of Lange *et al*. [41]. It contains 18 samples, 6 lean and 12 obese, with 674 lipids from 16 lipid classes. The comparison of mesenchymal stem cells (MSC) to adipogenic cells is coming from the supplement of Levental *et al*. [42]. Lipid species measured in less than 50% of all samples were removed before analysis with LINEX$^2$. The processed data contains 577 lipid species from 21 lipid classes and 4 samples per analyzed sample group (undifferentiated PM untreated and adipogenic PM untreated).

For all data sets analyzed with LINEX$^2$, HTML files with the LINEX$^2$ output are available at https://doi.org/10.6084/m9.figshare.20508870.

## Results

### A framework for lipid network creation and analysis

**Figure 1:**
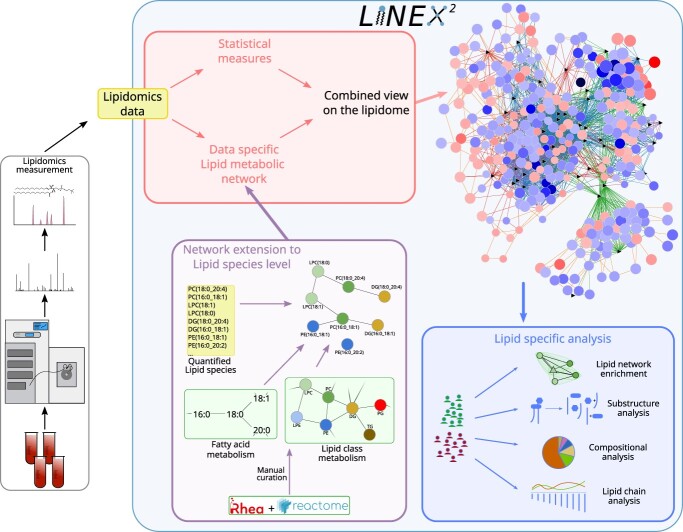
Lipidomics data are used as an input to LINEX$^2$. The lipids are then utilized to perform network extension that converts lipid class and fatty acid metabolic networks to lipid species, which are then visualized together with statistical measures such as t-tests or correlations. The network is also used as a basis for lipid substructure, compositional and lipid chain analysis. A lipid network enrichment algorithm, which takes enzymatic multispecificity into account, can be used to generate hypotheses for enzymatic dysregulation.

The workflow of a lipidomics experiment can be divided into five steps: sampling, sample preparation, data acquisition, data processing and data interpretation [43]. LINEX$^2$ is aiming at the biological interpretation of lipidomics data (Figure 1). The LINEX$^2$ builds data-specific lipid metabolic networks. To obtain these networks, we developed a network extension algorithm (Figure 1, purple box), where metabolic reactions on the lipid class level and fatty acid reactions are extended to the lipid species level. Network extension is possible with molecular species (e.g. DG(16:0_18:1)) or sum species data (e.g. DG(34:1)). Sum species are internally converted to molecular species, to incorporate modifications or additions/removals of fatty acids. This is achieved by finding sets of fatty acyls matching the sum composition using fatty acids commonly observed in lipid classes (e.g. DG(16:0_18:1), DG(16:1_18:0) or DG(14:0_20:1) for DG(34:1)). These can be adapted for each experimental setup. If molecular species are identified but not quantified they can be used instead of inferring fatty acyl sets, as reported in other studies [16]. An example for the network extension is the lipid class reaction between a Phosphatidylcholine (PC) and a Diacylglycerol (DG) (PC $\rightarrow $ DG), where the phosphocholine headgroup is cleaved off, is applied to the molecular lipid species PC(16:0_18:1) $\rightarrow $ DG(16:0_18:1) (for a detailed description see Materials & Methods section Network extension). Also, fatty acid reactions, such as elongation or desaturation can optionally be added to the network as heuristics, e.g. for Lyso-PC(18:0) (LPC(18:0)) $\rightarrow $ LPC(18:1). Since such reactions usually do not occur on complex lipids directly, but rather as activated fatty acids, they help to visualize fatty acid-specific effects on the network, as previously shown [19], and facilitate network analysis.

### Comprehensive curation of lipid-metabolic reactions

**Figure 2:**
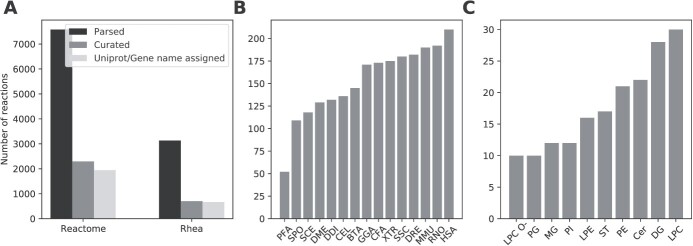
**(A)** Number of lipid-reactions parsed from Reactome and Rhea databases (black), after curation for available lipid classes and number of curated reactions (dark-gray), for which Uniprot or gene name annotations were available (light-gray). **(B)** Curated reactions per organism from the Reactome database (Rhea does not list details about organisms). Legend: HSA - *Homo sapiens*, RNO - *Rattus norvegicus*, MMU - *Mus musculus*, DRE - *Danio rerio*, SSC - *Sus scrofa*, XTR - *Xenopus tropicalis*, CFA - *Canis familiaris*, GGA - *Gallus gallus*, BTA - *Bos taurus*, CEL - *Caenorhabditis elegans*, DDI - *Dictyostelium discoideum*, DME - *Drosophila melanogaster*, SCE - *Saccharomyces cerevisiae*, SPO - *Schizosaccharomyces pombe*, PFA - *Plasmodium falciparum*. (**C)** Top 10 lipid classes with the most curated class reactions.

The basis for our network extension are publicly available metabolic reaction databases. To provide a comprehensive overview of lipid metabolism, we curated lipid class reactions from the Rhea [36] and Reactome [32] databases (Figure 2A). During curation, we removed all transport reactions and specialized modifications such as oxidations or fatty acid branching, which cannot be annotated to standardized lipid classes or are not generalizable for automated network extension. Curation resulted in over 3000 annotated reactions from both databases combined (Figure 2A) across organisms, including organism-specific reactions from Reactome. The top three organisms including the most reactions from Reactome are *H. sapiens* (HSA), *R. norvegicus* (RNO) and *M. musculus* (MMU) (Figure 2B). After database processing, LPC is the lipid class participating in most reactions (Figure 2C), followed by DG. All reaction identifiers are individually linked, providing a reference to the original database entries in the network.

To keep the freely available LINEX$^2$ software up-to-date, user contributions for new lipid classes and lipid-metabolic reactions can be made using an online form (https://exbio.wzw.tum.de/linex2). This way LINEX$^2$ can be updated in a community effort to enhance support for less studied parts of the lipidome.

### An approach to analyzing lipid networks

**Figure 3:**
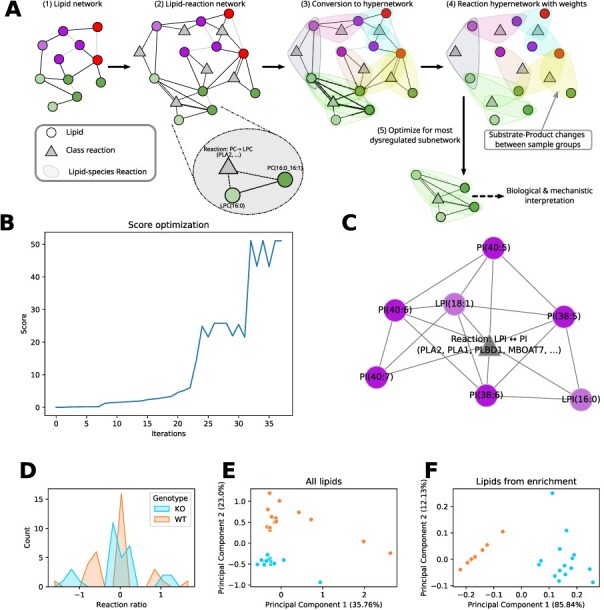
**(A)** Description of network enrichment workflow. In brief, the lipid network is converted into a hypernetwork, in which hyperedges correspond to lipid species reactions. Based on the computed dysregulation per hyperedge, an optimization algorithm finds the subnetwork with the maximum dysregulation. **(B)** Optimal subnetwork predicted by the enrichment algorithm for mice liver lipidomics data by [40]. The comparison is between wild-type and MBOAT7 knock-out samples. The resulting subnetwork shows the LPI $\leftrightarrow $ PI reaction at the center, surrounded by polyunsaturated PI species and two LPI species. **(C)** Progression of the objective function score during optimization that yielded the subnetwork in (B). **(D)** Substrate–product ratio distribution for the LPI $\leftrightarrow $ PI class reaction for all lipid species reactions per genotype (MBOAT7 deficient (KO) and wild type (WT)). **(E)** Principal component analysis of full lipidomics data and (**F)** of a subset of the lipidomics data containing only the lipids from the enriched subnetwork from (B). The color code is the same as in (D) for both plots.

**Figure 4:**
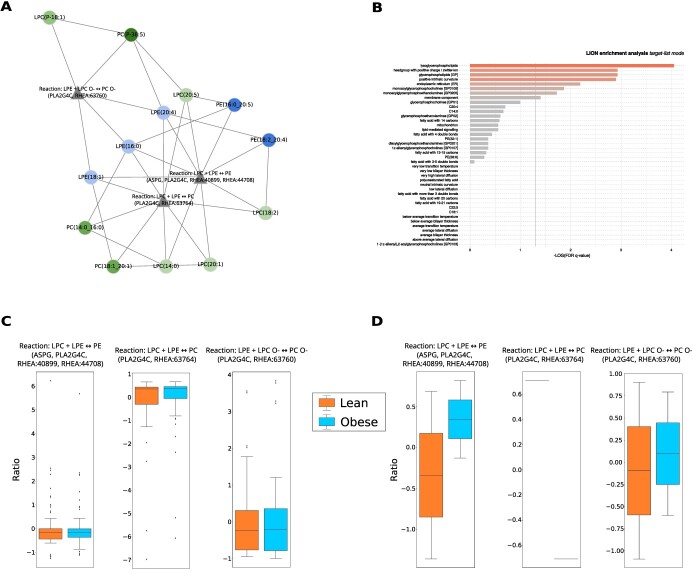
LINEX$^{2}$ application on the AdipoAtlas data. **(A)** Subnetwork returned by the introduced enrichment algorithm. The enriched subnetwork contains three reaction nodes, all representing fatty acid transfer between lysophospho- and phospholipids. Furthermore, the network shows a preference for long-chain polyunsaturated fatty acids. **(B)** LION enrichment using the lipids in the subnetwork (A) as targets in the target list mode. **(C)** Distribution of the substrate to product changes (see Methods - Substrate–product change calculation) for the three reactions present in A over all possible lipid species combinations from the AdipoAtlas data. **(D)** Distribution of the substrate to product changes using only the lipid species combinations identified in A. In both C and D ratios are shown as per-reaction *z*-scores.

For interpreting quantitative changes in molecular networks, network enrichment can be a powerful approach. In the context of metabolic or lipid networks, such methods can reveal underlying changes in enzymatic activity. In PPI networks, changes in protein abundances correspond directly to functional changes of the nodes, representing proteins, in the network. However, when analyzing (lipid-)metabolic networks enzymatic changes can only be approximated from changes in metabolite abundances between experimental conditions. In lipid-metabolic networks, an additional challenge comes from the multispecificity of involved enzymes. In LINEX$^2$-networks (as implemented in the network extension) every edge between two lipid species corresponds to an enzymatic reaction, therefore enzymes can correspond to multiple edges.

Our method is designed to explicitly take multispecificity into account. Therefore, a hypernetwork, establishing connections not only between lipids but also reactions, is required. Based on this representation, the enrichment algorithm can connect solutions from the same class reaction, promoting solutions explainable by a few metabolic reactions. Figure 3A shows the workflow of the enrichment analysis (for details see the Materials & Methods section Network enrichment). We start with a LINEX$^2$-network, where reactions are represented as edges (1). In the next step, we add lipid class reactions as a second type of nodes to the network (2). Edges between a class reaction node and all lipid species participating in this reaction are introduced, in addition to lipid–lipid edges, that represent conversions. This network is converted to a hypernetwork, where each hyperedge represents a lipid species reaction with lipid–substrates, –products and -reaction nodes (3). For each hyperedge (lipid species reaction), the dysregulation is quantified by the relative change of the lipid substrate–product ratio or difference between two experimental conditions (4). Considering both substrates and products is especially important for reversible reactions [44]. The reaction network is then used to find a maximally dysregulated subnetwork by employing a simulated annealing-supported local search (5). Heuristic reactions are penalized in the objective function of the network enrichment and serve only to increase connectivity. Additionally, the number of class reactions in the network can be penalized to favor parsimonious solutions with a simple mechanistic explanation.

### Inferring known enzymatic dysregulation from a knock-out study

As a proof of principle for the enrichment, we selected data from Thangapandi *et al*. [40]. In this study, the authors compared liver lipidomics of mice with a hepatospecific deficiency of MBOAT7 (KO) to wild-type (WT) mice under non-alcoholic fatty liver disease (NAFLD) condition. MBOAT7 catalyzes the class reaction fatty acyl-CoA + LPI $\rightarrow $ PI + CoA with a specific preference for Arachidonic acid (20:4($\omega $-6), AA) [45]. The data from Thangapandi *et al*. [40] are well suited for testing our enrichment algorithm because the enzymatic origin of lipidomic changes in liver tissue is known and the lipidome is affected by the disease.

Figure 3B shows the score progression during the optimization of the algorithm. The temporary plateau at a score of 25 shows the need for global approximation methods such as simulated annealing. In Figure 3C, the optimal subnetwork is shown (full interactive network available at https://doi.org/10.6084/m9.figshare.20508870). It consists only of PI, LPI species and one class reaction. This class reaction represents the transformation between LPI and PI. LINEX$^2$ cannot differentiate between the exact enzyme for this reaction. However, in contrast to e.g. PLA2, MBOAT7 only catalyzes LPI $\rightarrow $ PI class reactions. Additionally, MBOAT7 is known for a higher affinity for AA [45]. This preference can also be observed in the solution in Figure 3C for the edge between LPI(18:1) and PI(38:5), under the assumption that this reaction can only occur if the molecular composition of PI(38:5) is PI(18:1_20:4). Furthermore, all other reactions between LPIs and PIs are only possible for the addition/removal of fatty acyls with at least 20 carbon atoms and 4 double bonds. These results are not surprising, because of the structural similarity of AA to other (very)-long-chain polyunsaturated fatty acids (Supplementary Figure S3). A recently published preprint by [46] elucidated the structure and catalytic mechanism of MBOAT7 and found that saturated acyl-CoAs are less likely to bind, supporting our results. To further showcase how our method prefers the right fatty acid preference, we plotted the distribution of all lipid species for MBOAT7 (Supplementary Figure S4A) and the same distributions for only those lipid species reactions in the subnetwork (Supplementary Figure S4B). While the former plot shows very similar distributions between WT and KO, the selected species plot clearly shows that while the WT reactions are staying close to zero, the KO distributions show high absolute ratios in a bimodal fashion.

While LINEX$^2$ is not able to directly pinpoint MBOAT7, the results demonstrate its capability to find strong hypotheses for enzymatic dysregulation from lipidomics data. To evaluate the enrichment results, we implemented an empirical *P*-value estimation procedure (detailed description in Materials & Methods section Network enrichment). The MBOAT7 enrichment result (Figure 3C) has a *P*-value of 0.0018, indicating the likeliness of the mechanistic solution.

When investigating the distributions of the LPI $\leftrightarrow $ PI class reaction (i.e. over all respective lipid species reactions) per genotype (Figure 3D), no strong distribution shift in one direction can be observed. The distributions show a peak around zero, indicating that many reaction ratios are not influenced by the MBOAT7 knock-out (KO). However, two more peaks around 1 and –1 can be observed for both conditions, where the peaks of the KO are shifted slightly more towards absolutely higher values. Despite these subtle differences, it is not possible to draw a hypothesis towards a mechanistic explanation including fatty acid-specific effects. In Figure 3E, we plotted the principal component analysis (PCA) of the full lipidomics data. In contrast, Figure 3F shows the PCA plot based only on the lipidomics data for the lipid species present in the enrichment subnetwork (Figure 3C). In the PCA of all lipids, PC2 reflects the variance corresponding to the genotype, explaining 23% of the total variance. However, after selecting the LPI and PI species from the enrichment solution, the genotypic difference makes up for the majority of the variance with almost 86%. This means that the lipids in the subnetwork (Figure 3C) represent the effect of the MBOAT7 knock-out almost entirely.

These results demonstrate the ability of the enrichment analysis to develop reasonable hypotheses on enzymatic dysregulation based on lipidomics data. The result not only shows an increased variance corresponding to the genotype but also allows mechanistic lipid species-specific explanations.

### A mechanistic hypothesis for adipocyte expansion in obesity

We further aimed at improving our understanding of the changes in lipid-metabolism of lipid-related diseases. For this purpose, we selected the AdipoAtlas [41], a reference lipidome of adipose tissue in lean and obese humans. The authors identified 1636 molecular lipid species, out of which 737 were quantified.

#### Network analysis indicates a mechanism for adipocyte expansion

We used our network enrichment algorithm, which resulted in the subnetwork shown in Figure 4A. The subnetwork contains three reactions, which all represent an acyl-transferase reaction between Lyso-Phospholipids. Investigating the reaction ratios of these three class reactions over all possible species reactions shows equal distributions between obese and lean (Figure 4C). However, considering the species reactions present in the subnetwork reveals differences between the groups with respect to the reaction ratios (Figure 4D). These reactions are catalyzed by the Phospholipase A2 Group IVC (PLA2G4C) and the asparaginase (ASPG), which both have lipase and acyl-transferase activity. It has been shown that PLA2 Group IV members preferably act on the sn-2 position and that polyunsaturated fatty-acyls are commonly transferred by them [47]. This preference is reflected in the subnetwork. Literature research shows that PLA2G4C has been reported to be differentially expressed in obese individuals [48, 49] and products of (c) PLA2 activity are known mediators of adipose tissue metabolism [50].

**Figure 5:**
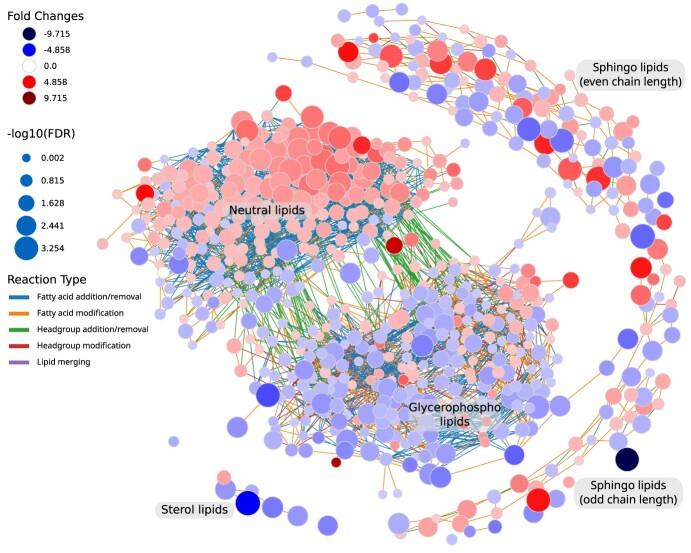
Lipidomics data from the AdipoAtlas visualized with LINEX. In the network lipids are represented as circular nodes. The red color of lipid nodes represents a positive fold change from lean to obese condition, and blue a negative fold change. Edge color indicates the type of reaction connecting two nodes. An interactive version of the network as well as all other analyses conducted with LINEX are available in an HTML file at https://doi.org/10.6084/m9.figshare.20508870.

The prevalence of acyl-transferase reactions in the subnetwork suggests a transfer of FAs between lipids with a Phosphocholine and a Phosphoethanolamine headgroup and their respective Lyso-Phospholipid species. The ratio of LPC/LPE to PC/PE as well as the ratio of lipids with a Phosphocholine headgroup to lipids with a Phosphoethanolamine headgroup influences the membrane curvature [51, 52]. This property is important because adipocytes expand in obesity [53]. A change in this ratio has also been associated with altered membrane integrity and fluidity [54, 55]. We confirmed this with a Lipid Ontology (LION) enrichment analysis [33], where we used the lipids of the enriched subnetworks as a target list (Figure 4B). The analysis resulted in membrane curvature and other membrane-related terms. Additionally, we observed similar behavior in the development of mesenchymal stem cells to adipogenic cells based on data from [42] (Supplementary Figure S5B). These insights further support the practical feasibility of our reaction enrichment approach.

#### Lipid moieties show alterations in neutral lipid composition

Despite changes in the Glycerophospholipid composition that are an indication for adipocyte expansion, synthesis and accumulation of neutral storage lipids is a major hallmark for obesity. This is also reflected in the network representation of the AdipoAtlas lipidome (Figure 5). It shows increased TG and DG levels in obese samples, and an overall decrease in Glycerophospholipids. Neutral lipid species containing poly-unsaturated FAs have especially high fold changes (Figure 5, Supplementary Figure S6). Concerning chain length, we observe that TG species with a sum length >30 and <57 are accumulated in obese samples (Supplementary Figure S7A). Since TGs and DGs are synthesized de-novo, they were not picked up by the network enrichment as strong alteration between lipid classes, we wanted to further investigate the compositional changes of neutral lipids. For this, we developed a lipid moiety analysis. It quantifies common substructures of lipids across the lipidome to show trends in changes of the lipidome composition (Supplementary Figure S2). Especially lipid species with a sum length >45 and 2–3 double bonds show a sharp increase in obesity, predominantly TG species with a length of 49 and 53 (Supplementary Figure S7B). Also Sterol esters show significant changes in disease progression. The observed changes in the TG composition are in accordance with previously published results [56]. This analysis can provide additional insights into the lipid metabolism and complement the network analysis.

### LINEX$^2$ software

The LINEX$^2$ software framework for analysis and visualization of lipid networks is available as a web service at https://exbio.wzw.tum.de/linex2. Lipidomics data can be used to perform not only network enrichment and visualization, but also summarizing statistics, lipid chain analysis [57], and moiety analysis. Results can be viewed and downloaded in an interactive format. For high-throughput analysis, a python package is also available (https://pypi.org/project/linex2/). The details of the implementation can be found in the Supplementary Materials.

## Discussion

We present a method to generate and analyze lipid-metabolic networks. Using curated lipid class reactions from common metabolic databases our method computes data-specific lipid networks. Furthermore, we developed a network enrichment algorithm, to propose hypotheses for enzymatic dysregulation from lipidomics data. As a proof of principle, we applied the approach to liver lipidomics data, where the deficient MBOAT7 enzyme was successfully identified from the data.

The challenge in generating mechanistic hypothesis from metabolomics or lipidomics data lies in the fact that dysregulation on the enzymatic level is not measured directly. Instead it can only be inferred based on changes in the metabolome, unless full-scale proteomics experiments are run in addition. For lipid networks, only one tool, BioPAN, is available so far [20]. In contrast to our proposed network enrichment algorithm, this method is searching for activated reaction chains between lipids of the same sum composition. The scope of the LINEX$^2$ enrichment differs from BioPAN, by searching for dysregulation of multispecific enzymes that likely affect lipids of the same class with different sets of fatty acyls. Another difference is in the network computation. LINEX$^2$ includes fatty acyl addition/removal, enabling insights such as the MBOAT7 example we show in this work. To illustrate how LINEX$^2$ compares to BioPAN [20], we computed the BioPAN network (Supplementary Figure S8A) as well as the predicted list of active reactions. The results do not include LPI species and only one reaction chain with a PI species (Supplementary Figure S8B). Therefore a hypothesis on MBOAT7 dysregulation cannot be drawn from this method. Similarly for the application of Shiny GATOM [30] on the same data (Supplementary Figure S9). The subnetwork contains many reactions but misses reactions of PI with more than 40 carbon atoms and does not attribute reactions between PI and LPI to MBOAT7. [21] performed a network optimization based on changes in lipid abundances and literature mining of lipid–enzyme interactions. However, they do not infer quantitative values for reactions and no implementation is available. Hence, LINEX$^2$ lipid network enrichment is the only available method that aims at inferring enzymatic dysregulation from lipidomics data. An important aspect of the method is the usage of hypernetworks, to take the multispecificity of lipid enzymes into account, which increases confidence in the retrieved mechanism.

A limitation of our enrichment algorithm is that it computes substrate–product ratios independent from each other. In reality, however, reactions are linked through shared substrates or products and metabolic changes are propagated through the network. These effects can be due to, e.g. metabolic self-regulation [58] and structural or signaling functions. Since each lipid species takes part in a plethora of reactions, results of altered enzymatic activity might not be observed directly for the substrates and products of that reaction. This is also the case for multiple reactions, which form a consecutive transformation sequence that change at the same time. However, assuming the principle of maximum parsimony, disordered conditions are most likely caused by alterations in only a few enzymatic steps, making the settings for such inaccurate approximations rare cases. Our network extension method depends on generalizable reaction rules. Therefore, manual curation of reaction databases was necessary. Due to a better coverage of commonly measured lipid classes, metabolic databases may be susceptible to research bias. We address this bias by using lipid class reactions instead of enzymes, to prevent well-studied enzymes participating in many reactions from being favorably selected. Additionally, the network enrichment is avoiding bias by correcting for the number of lipid participants in the reaction. Our method is constrained to returning a set of candidate enzymes, which are attributed to the same type of reaction, without pinpointing individual enzymes. With more data available, such as the work from Hayashi *et al*. [47], better estimates for fatty acid-specific subnetworks can be made.

With the ability to connect enzymatic activity to lipidomics data, LINEX$^2$ provides the basis for a knowledge-driven integration of lipidomics with proteomics data. The inclusion of quantitative proteome information could further improve the performance of the enrichment algorithm presented in this paper and open up the possibility of directly identifying causal proteins. This could be of great value for the causal interpretation of lipidome changes, which would directly translate into relevance for clinical applications, due to the many associations of lipids with various disorders [7, 8, 13, 16, 49].

With our LINEX$^2$ web service, we offer new analysis methods for lipidomic data, ranging from network visualization to generating hypotheses for dysregulation. Freely available through a user-friendly interface, lipidomics researchers do not need to be experts in bioinformatics to perform sophisticated analyses of the lipidome in a metabolic context. Moreover, LINEX$^2$ networks can be the basis for further methodological developments that help to enhance the biological interpretability of lipidomics experiments by enabling inference of metabolic regulation from lipid data.

**Key Points**

- Data-specific lipid networks are computed based on reactions from the Rhea and Reactome databases.
- A novel enrichment method identifies enzymatic dysregulation in custom lipidomics datasets.
- Moiety analysis elucidates relevant lipid structural features contributing to dysregulation.
- We apply the approach on clinically relevant lipidomics data to generate mechanistic hypotheses adipocyte expansion.
- LINEX$^2$ is freely available as a web service at https://exbio.wzw.tum.de/linex2.

## Data availability statement

LINEX is free software. Source code: GitLab (aGPLv3 License): https://gitlab.lrz.de/lipitum-projects/linex

Figure reproducibility: https://gitlab.lrz.de/lipitum-projects/LINEX2-paper-code

ALEX123 lipid classes and curated database reactions: https://gitlab.lrz.de/lipitum-projects/LINEX2_package/-/tree/master/LINEX2/data

## Authors’ contributions

J.K.P. supervised the project and secured the funding. N.K., T.D.R. and J.K.P. planned and conceptualized the work. N.K. and T.D.R. developed the web service. N.K., O.E.L. and T.D.R. designed and implemented the network enrichment procedure. L.F., L.K. and T.D.R. parsed and curated the reaction databases, and implemented the network extension. N.K. and T.D.R. applied, validated and interpreted the approach on lipidomics data. N.K., O.E.L., T.D.R. and J.K.P. wrote the manuscript. All authors read, reviewed and accepted the manuscript in its final form.

## Supplementary Material

LINEX2_BiB_revision_Supplementary_bbac572

SupplementaryTableS1_bbac572

## Author Biographies

**Tim D. Rose** was a PhD student in the research group LipiTUM, TUM School of Life Sciences, Technical University of Munich. He is currently a postdoctoral fellow at the European Molecular Biology Laboratory (EMBL).

**Nikolai Köhler** is a PhD student in the research group LipiTUM, TUM School of Life Sciences, Technical University of Munich.

**Lisa Falk ** was a master student in the research group LipiTUM in the program  Molecular Biotechnology at the Technical University of Munich.

**Lucie Klischat** was a bachelor student in the research group LipiTUM in the program Bioinformatics at the Technical University of Munich

**Olga E. Lazareva** was a PhD student in the Chair of Experimental Bioinformatics at the TUM School of Life Sciences, Technical University of Munich. She is currently a postdoctoral fellow at the German Cancer Research Center (DKFZ) and the European Molecular Biology Laboratory (EMBL).

**Josch K. Pauling** is a group leader of the research group LipiTUM at the TUM School of Life Sciences, Technical University of Munich. He obtained his PhD from the University of Southern Denmark.
