# Supplementary material for: Lipid network and moiety analysis for revealing enzymatic dysregulation and mechanistic alterations from lipidomics data
Source: Brief Bioinform. 2023 Jan 2;24(1):bbac572. doi: 10.1093/bib/bbac572 (PMC9851308; doi:10.1093/bib/bbac572)
Supplement: LINEX2_BiB_revision_Supplementary_bbac572 [file linex2_bib_revision_supplementary_bbac572.pdf]

Lipid network and moiety analysis for revealing enzymatic dysregulation and mechanistic alterations from lipidomics data

Supplementary Methods

Network Extension

Extension of class metabolism Lipid class reactions are evaluated using the defined reaction categories (headgroup removal/addition, headgroup modification, fatty acid addition/removal, and lipid merging) plus ether heuristic. For each reaction, all lipids from the user data, which match the lipid classes that participate in a reaction are selected. Reactions with more than one lipid class as substrate and product are only possible or available for certain reaction categories. If possible, these are explicitly mentioned. The reaction evaluations are under the condition that a lipid class reaction for the substrate-product set exists. For a "Headgroup modification" reaction the substrate and product lipids require the same set of fatty acids, e.g. PS(18:0_16:0) ↔ PE(18:0_16:0, (Supplementary Figure S1A). A "Headgroup addition/removal" also requires the substrate and product lipids to have the same set of fatty acids, e.g. DG(18:0_18:1) ↔ PA(18:0_18:1) (Supplementary Figure S1B). The reaction is also possible for two lipids as substrates and two lipids as products, e.g. PC + Cer ↔ DG + SM (Supplementary Figure S1C). In this case, the headgroup is shifted from one lipid to another. For this evaluation, two substrate product pairs are matched for the lipid donating the headgroup (PC ↔ DG) and the lipid accepting it (Cer ↔ SM). These are then evaluated independently if at least one reaction per pair can be found. The "Fatty acid addition/removal" reactions in the case of one lipid as substrate and product require one lipid with one less fatty acid and the fatty acids of the lipid with fewer fatty acids to be contained in the other lipid, e.g. DG(18:0_18:1) ↔ TG(18:0_18:1_16:0) (Supplementary Figure S1D). For two substrates and two products, a fatty acid is shifted from one lipid to another, e.g. PE + MLCL ↔ CL + LPE. Again, two substrate product pairs are matched for the lipid donating the fatty acid (PC ↔ LPC) and the lipid accepting it (MLCL ↔ CL). They are evaluated independently and the edges are added to the network if two pairs can be found which donate/accept the same fatty acid. Another case exists for reactions with two substrates and one product (e.g. LPC + LPC ↔ PC). Also here, a fatty acid is shifted from one lipid to another, however, the donor is not considered a lipid, after the fatty acid is removed. Similarly, a pair of lipids accepting the fatty acid is formed (LPC ↔ PC). Edges are then added to the network if the accepting and donating lipids have combined the same fatty acids as the resulting lipid. The reaction type "Lipid merging" describes two lipids that are bound together by a reaction, e.g. PG + PA ↔ CL (Supplementary Figure S1E). The molecular species of the substrates require the same combined fatty acids as the resulting lipid for this reaction to occur and be added to the network. We additionally consider fatty acid ether exchange as heuristics. These optional connections are edges between lipid classes and their corresponding ether classes if they share the same set of fatty acids, e.g. LPA(18:1) ↔ LPA(O-18:1), to improve network connectivity and stress fatty acid-specific effects in the network. Edges in the network are undirected since we cannot conclude the net flux of a reaction from the lipidomics data, especially since for most reactions, counterparts in the opposite direction exist.

Extension of fatty acid metabolism Fatty acid synthesis and modification occurs commonly on activated fatty acids and they are not bound to complex lipids. However, to increase network connectivity, fatty acid reactions on complex lipids can be added to the network. As described earlier, this is done through user-defined reactions. A fatty acid reaction, e.g. PC(18:0_16:0) → PC(18:1_16:0), here fatty acid desaturation for the fatty acid 18:0 to 18:1, requires two lipids of the same lipid class and all but one identical fatty acid. Only one fatty acid modification is considered per reaction. In the case of elongation, the non-identical fatty acids require the same amount of double bonds, and other modifications have to differ by the length of two carbon atoms, e.g. 18:0 - 20:0. A desaturation requires fatty acids, which differ by a double bond, with all other attributes being the same. Custom fatty acid metabolism rules can be added, by providing the numeric changes of fatty acid attributes, such as length, double bonds, or modifications. Additionally, reactions between two specific fatty acids can be excluded. For example, the desaturation of fatty acyl 18:2 to 18:3 is not possible in humans.

In the network representation lipids are shown in the provided resolution. In the case of sum species, lipid nodes can also be shown as molecular species (based on the possible molecular species, as

explained earlier). Sum species including their statistical properties are then projected onto multiple potential molecular species.

Webtool Implementation

The web service is built with the Django web framework (`https://www.djangoproject.com/`) in the python programming language (version 3.8, `https://www.python.org/`). PostgreSQL (`https://www.postgresql.org/`) is used as a back-end database for Django, to store data, networks, and all computed attributes. Cookies are used to connect a browser session to uploaded data, their corresponding computed networks, and analyses. For interactive network visualizations, vis-network [1] is used and other interactive plots are done with Plotly [2]. All other user-site functionalities are implemented in plain JavaScript. PDF versions of networks are generated with the NetworkX package [3] in conjunction with the matplotlib library [4]. The backend was implemented in python. To achieve compatibility across operating systems, LINEX^2 can be built in a Docker environment. In the public LINEX^2 version, uploaded user data is temporarily stored on our server for a certain time or can be deleted manually by the user (for further information see `https://exbio.wzw.tum.de/linex/request-data-delete`). However, using the provided Dockerfiles LINEX^2 can also be easily run locally on any computer (for instructions check the source code repository). LINEX^2 is free software, published under the aGPLv3 license. The source code is available at `https://gitlab.lrz.de/lipitum-projects/linex`. While we adapted the procedure to generate lipid species networks, the original LINEX version can still be accessed through the website (marked as version 1).

Identified and quantified lipidomics data with optional sample labels can be uploaded to LINEX^2. Lipidomics data must be uploaded as a table with samples, lipids, and their corresponding concentrations/amounts. To convert lipids into our internal programming model, we recommend the LIPID MAPS nomenclature [5]. However, we integrated the LipidLynxX [6] software, which can convert multiple lipid nomenclatures, increasing the compatibility of LINEX^2 with multiple formats. A tutorial is available on the website (`https://exbio.wzw.tum.de/linex/tutorial`).

Statistical measures

To enable a combined visualization of the biochemical connections between lipid species and quantitative lipidomics measurements, LINEX^2 offers the possibility to project different statistical metrics onto the species networks.

To characterize the changes in lipid levels between different experimental conditions, we provide unpaired parametric (t-test), non-parametric (Wilcoxon rank-sum test) test options, and a paired parametric test (Wilcoxon signed-rank test). All resulting p-values are automatically false-discovery rate (FDR) corrected using the Benjamini-Hochberg procedure [7]. Furthermore, fold changes are computed to showcase effect size. For the computation of these metrics, we used the scipy package [7, 8] in conjunction with the statsmodels package [9]. All measures can be visualized either as node sizes or node colors.

Measures for lipid connections (i.e. edges in the network) are correlation-based. Specifically, the options provided are spearman's correlation and partial correlation. All correlations above a user-specified significance threshold (default = 0.05) are set to 0 automatically. Correlation values can be visualized in the network representation through edge colors.

Network enrichment p-value

In the implementation for the LINEX^2 web service, the local search is run multiple times (default=5), each with random seeds. The best result can then be optionally used as a seed for another local search run, to improve this result. The best score achieved in all local search runs is then returned to the user.

Lipid chain analysis

We implemented lipid chain analysis in python according to the proposed method by Mohamed, Molendijk, and Hill [10]. For each lipid class, lipid species with the same sum length of fatty acids are summed up per sample and a mean over all samples of one experimental condition is calculated. After that, the fold change between a selected control and e.g. a disease condition is calculated for

each sum length per lipid class. The result is then plotted with an ascending fatty acid length on the x-axis, showing class-specific fatty acid length fold changes between conditions.

Supplementary Figures

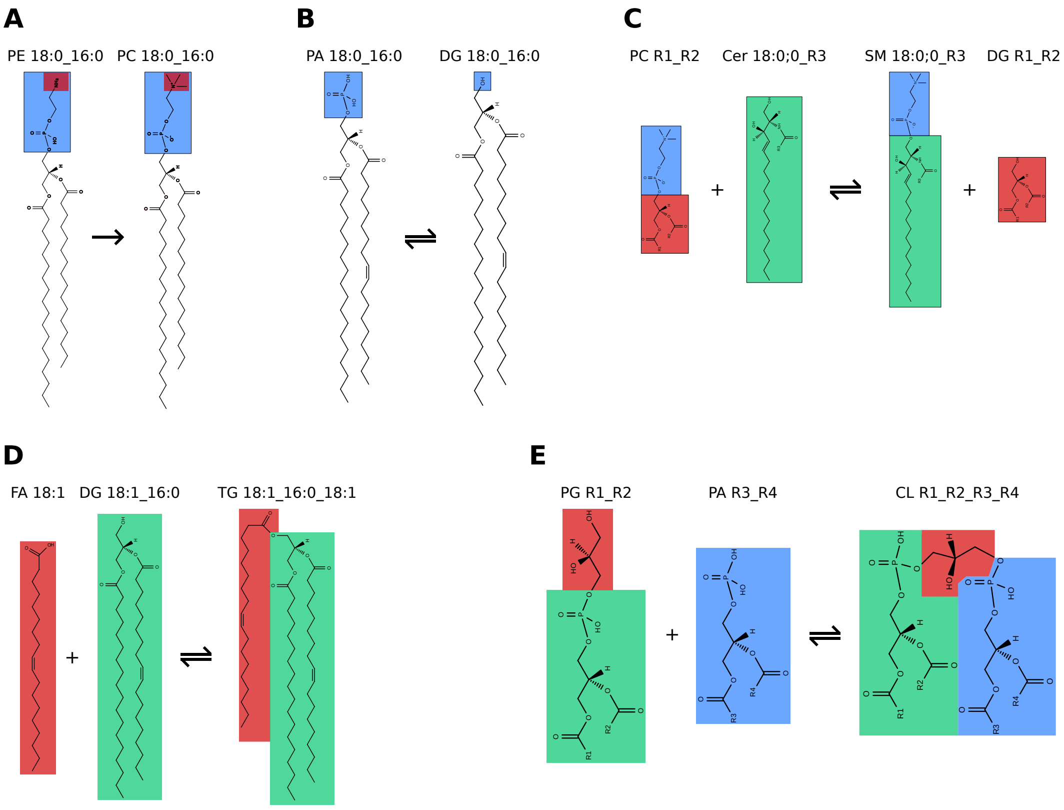

Figure S1: Schematic depiction of common biochemical lipid modifications/transformations. **A** Tri-methylation of the ethanolamine moiety of a PE produces the corresponding (i.e. same fatty acid combination) PC species **B** De-phosphorylation of a PA transforms it into a DG. **C** The phosphocholine group of PC is transferred onto a Cer, the PC-corresponding DG and the Cer-corresponding SM species. **D** Esterification of a free fatty acid onto a DG produces a TG. **E** PG and PA are linked via esterification of the glycerol and the phosphate group. The Cardiolipin product contains the same fatty acid residues as the substrates.

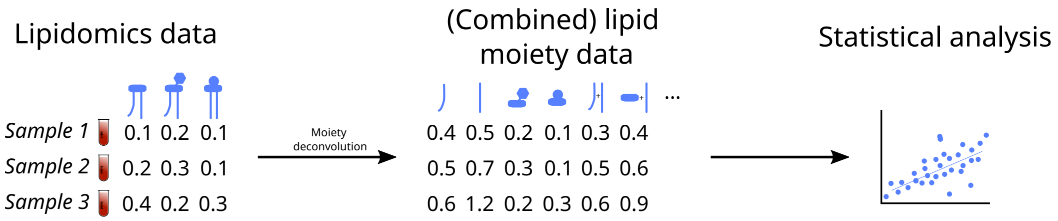

Figure S2: Workflow of lipid moiety analysis. Lipids from lipidomics data are deconvoluted into quantitative moieties. Moiety data is then used for statistical analysis, such as regression.

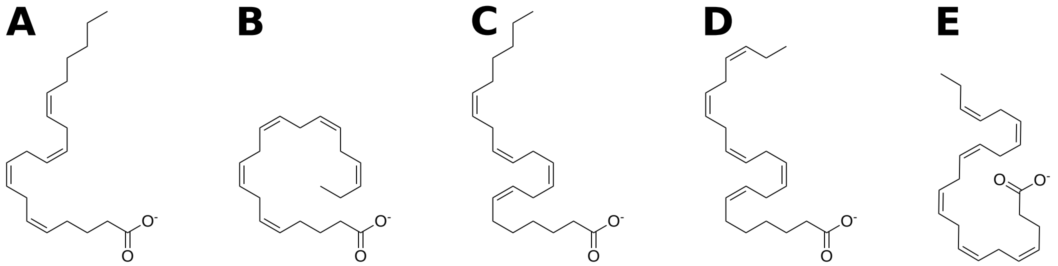

Figure S3: Fatty acid candidates for the acyltransferase reaction catalyzed by MBOAT7 predicted by the enrichment shown in Figure 4. **A** (5Z,8Z,11Z,14Z)-eicosatetraenoate (SwissLipids ID: SLM:000000296), **B** (5Z,8Z,11Z,14Z,17Z)-eicosapentaenoate (SwissLipids ID: SLM:000000409), **C** (7Z,10Z,13Z,16Z)-docosatetraenoate (SwissLipids ID: SLM:000001124), **D** (7Z,10Z,13Z,16Z,19Z)-docosapentaenoate (SwissLipids ID: SLM:000000929), and **E** (4Z,7Z,10Z,13Z,16Z,19Z)-docosahexaenoate (SwissLipids ID: SLM:000001084).

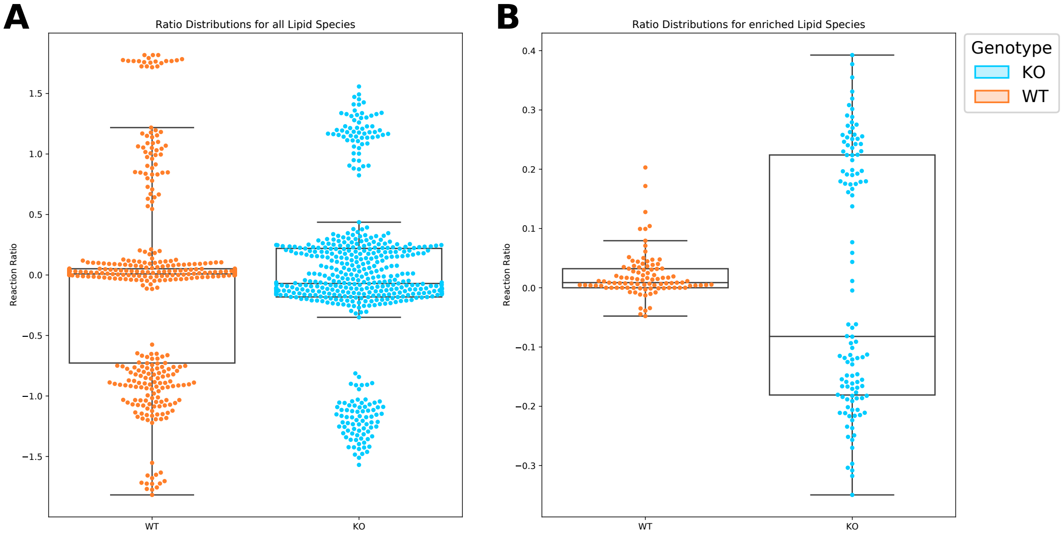

Figure S4: Distribution of MBOAT7 reaction ratios per sample group. **A** Ratios for a lipid species reactions found in the MBOAT7 dataset. **B** Ratios for all lipid species reactions extracted during the enrichment step.

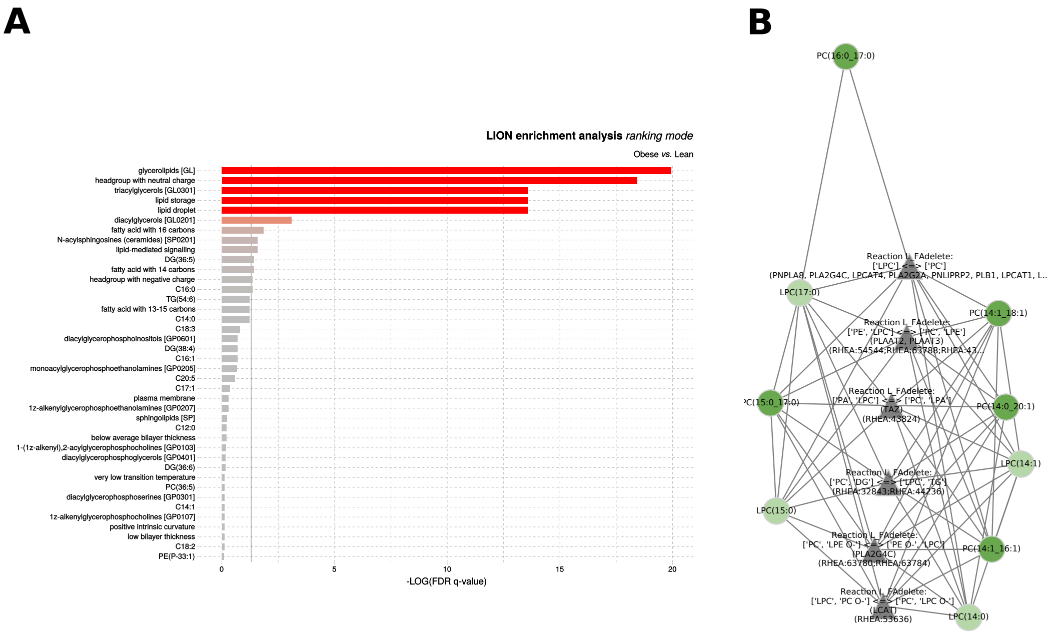

Figure S5: **A** LION enrichment [11] for the AdipoAtlas data with all lipids in the enrichment subnetwork (Main Figure 4A) as LION enrichment based on p-values from a student's t-test. **B** Enrichment result of the analysis of cell culture data comparing mesenchymal stem cells and adipogenic cells. The subnetwork only comprises lipids with a Phosphocholine head group.

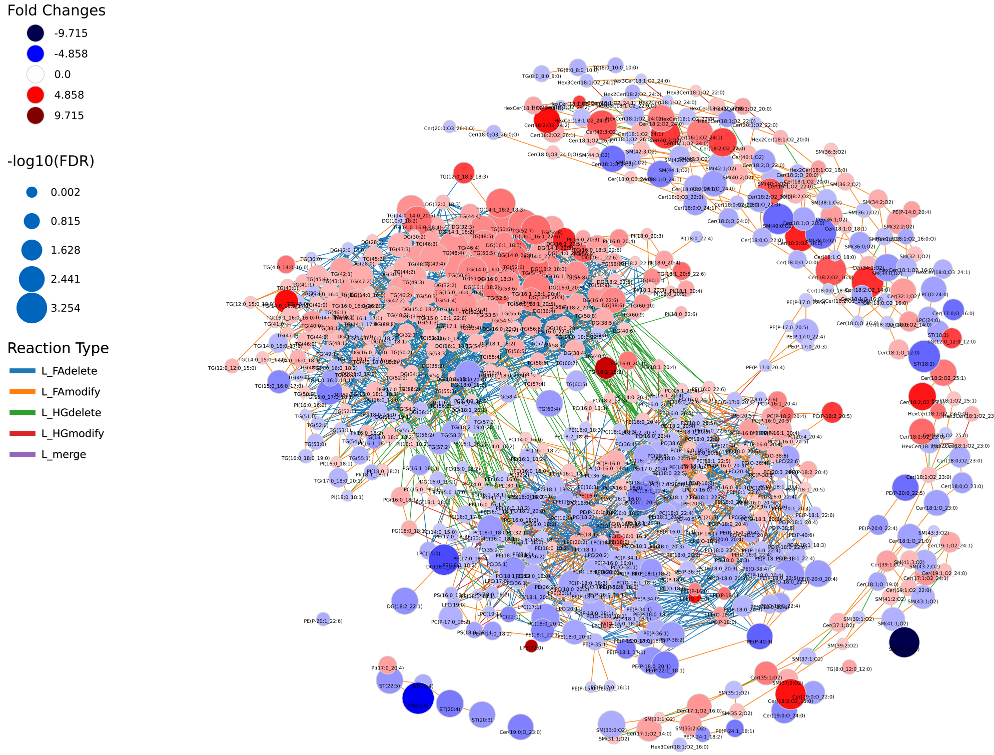

Figure S6: Annotated AdipoAtlas network. For a description of the network see Figure 4 and the Results section.

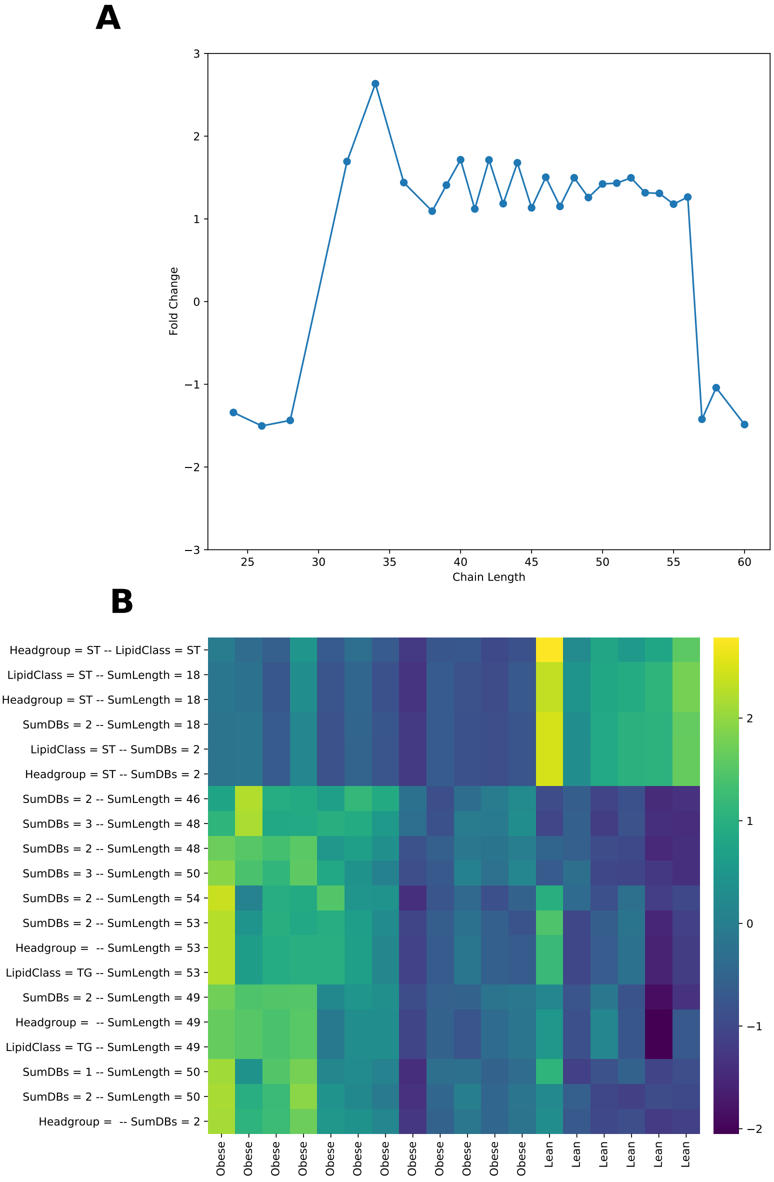

Figure S7: Additional results of LINEX^2 analyses on the AdipoAtlas data. **A** Results of the chain length analysis showing fold changes between obese and lean samples for the sum of all TG species per sum length. The chain length analysis is explained in the Supplementary Methods. **B** Substructure analysis results, showing the 20 best property combinations. Features were selected by their absolute coefficients in a regression analysis with the obesity state (i.e. obese or lean) as the target variable. Selected properties include steryl esters, which show lower values in obese samples, and sum length combinations between 46 and 54 only found in TG species in the AdipoAtlas data.

A

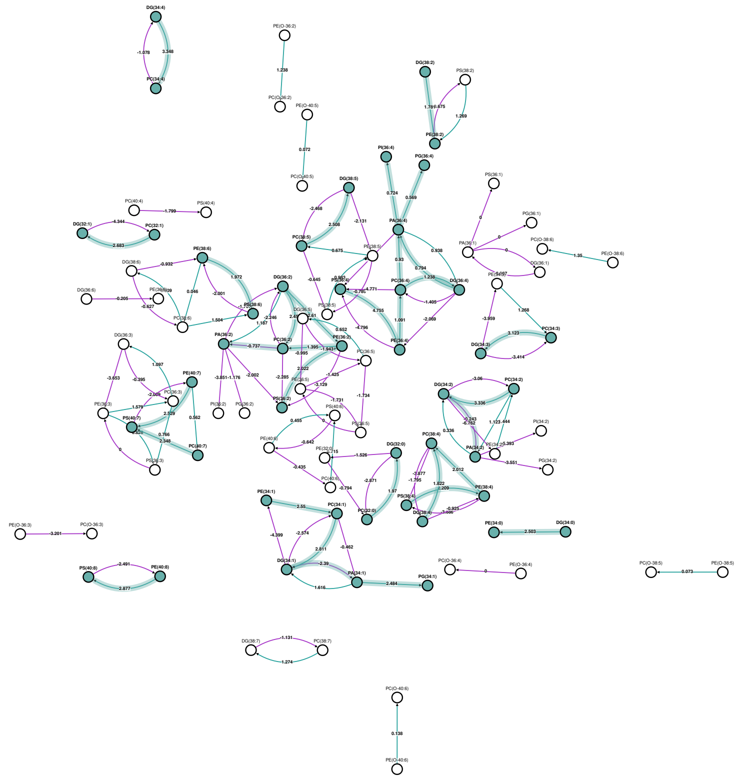

B

lipid species active reaction (WTHFCD vs KOHFCD)

Reactions chains	Z-score	Predicted genes
PS(36:4) → PE(36:4) → PC(36:4) → PA(36:4)	3.912	*PISD*, *PEMT*, *PLD1*, *PLD2*
PS(36:4) → PE(36:4) → PC(36:4) → DG(36:4) → PA(36:4) → PI(36:4)	3.847	*PISD*, *PEMT*, *DGKA*, *DGKB*, *DGKD*, *DGKE*, *DGKG*, *DGKH*, *DGKI*, *DGKK*, *DGKQ*, *DGKZ*, *CDS1*, *CDS2*, *CDIPT*
PS(36:4) → PE(36:4) → PC(36:4) → DG(36:4) → PA(36:4) → PG(36:4)	3.778	*PISD*, *PEMT*, *DGKA*, *DGKB*, *DGKD*, *DGKE*, *DGKG*, *DGKH*, *DGKI*, *DGKK*, *DGKQ*, *DGKZ*, *CDS1*, *CDS2*, *PTPMT1*
DG(34:4) → PC(34:4)	3.348	*CHPT1*
PC(34:3) → DG(34:3)	3.123	No genes have yet been identified
PS(38:4) → PE(38:4) → PC(38:4)	2.985	*PISD*, *PEMT*
PE(40:8) → PS(40:8)	2.877	*PTDSS2*
PC(34:1) → DG(34:1)	2.811	No genes have yet been identified
PE(34:1) → PC(34:1) → DG(34:1) → PA(34:1) → PG(34:1)	2.728	*PEMT*, *DGKA*, *DGKB*, *DGKD*, *DGKE*, *DGKG*, *DGKH*, *DGKI*, *DGKK*, *DGKQ*, *DGKZ*, *CDS1*, *CDS2*, *PTPMT1*
PC(32:1) → DG(32:1)	2.683	No genes have yet been identified
PE(40:7) → PS(40:7)	2.529	*PTDSS2*
PC(38:5) → DG(38:5)	2.508	No genes have yet been identified
DG(34:0) → PE(34:0)	2.503	*CEPT1*
PA(34:1) → PG(34:1)	2.484	*CDS1*, *CDS2*, *PTPMT1*
DG(36:2) → PC(36:2)	2.456	*CHPT1*
PS(36:2) → PE(36:2) → PC(36:2)	2.416	*PISD*, *PEMT*
PC(40:7) → PS(40:7)	2.348	*PTDSS1*
PC(34:2) → DG(34:2) → PA(34:2)	2.187	*DGKA*, *DGKB*, *DGKD*, *DGKE*, *DGKG*, *DGKH*, *DGKI*, *DGKK*, *DGKQ*, *DGKZ*
PE(38:4) → PC(38:4)	2.012	*PEMT*
PE(38:6) → PS(38:6)	1.972	*PTDSS2*
PC(32:0) → DG(32:0)	1.97	No genes have yet been identified
DG(36:2) → PE(36:2) → PC(36:2) → PA(36:2)	1.887	*CEPT1*, *PEMT*, *PLD1*, *PLD2*
DG(38:4) → PC(38:4)	1.822	*CHPT1*
DG(38:2) → PE(38:2)	1.781	*CEPT1*

Figure S8: BioPAN [12] results for the MBOAT7 knock-out data. **A** Computed lipid species network with z-scores for active reactions. **B** Predicted active lipid species reactions with corresponding z-scores and predicted genes.

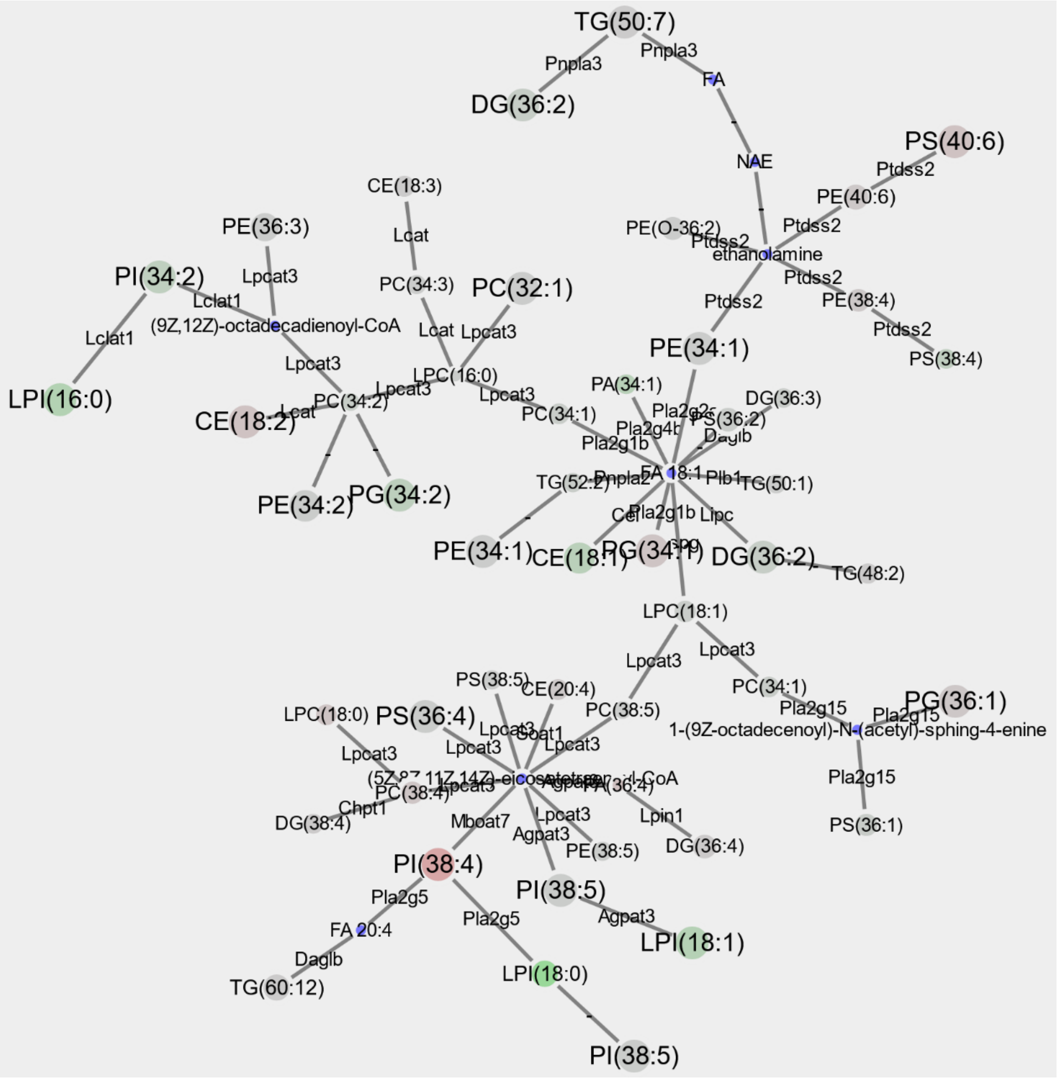

Figure S9: Shiny GATOM [13] results for the MBOAT7 knock-out data. Analysis was performed by differential analysis of the WT and MBOAT7 KO mice under the HFCD condition using the Rhea lipid database. The network shows the maximum weight connected subgraph as predicted by Shiny GATOM.

References

[1] Giancarlo Perrone, Jose Unpingco, and Haw-Minn Lu. "Network visualizations with Pyvis and VisJS". In: *arXiv preprint arXiv:1802.03426* (June 2020). arXiv: `2006.04951 [cs.SI]`.

[2] Plotly Technologies Inc. "Collaborative data science". In: *Montréal, QC* (2015).

[3] Aric A Hagberg, Daniel A Schult, and Pieter J Swart. "Exploring Network Structure, Dynamics, and Function Using Networkx". en. In: *Proceedings of the 7th Python in Science Conference (SciPy2008)*. Pasadena, CA USA, 2008, pp. 11–15.

[4] John D Hunter. *Matplotlib: A 2D Graphics Environment*. 2007.

[5] Eoin Fahy et al. "LIPID MAPS online tools for lipid research". en. In: *Nucleic Acids Res.* 35.Web Server issue (July 2007), W606–12.

[6] Zhixu Ni and Maria Fedorova. "LipidLynxX: a data transfer hub to support integration of large scale lipidomics datasets". In: *bioRxiv* 2020.04.09.033894 (2020).

[7] Yoav Benjamini and Yosef Hochberg. "Controlling the false discovery rate: A practical and powerful approach to multiple testing". en. In: *J. R. Stat. Soc.* 57.1 (Jan. 1995), pp. 289–300.

[8] Pauli Virtanen et al. "SciPy 1.0: fundamental algorithms for scientific computing in Python". en. In: *Nat. Methods* 17.3 (Mar. 2020), pp. 261–272.

[9] Skipper Seabold and Josef Perktold. *Statsmodels: Econometric and Statistical Modeling with Python*. 2010.

[10] Ahmed Mohamed, Jeffrey Molendijk, and Michelle M Hill. "lipidr: A Software Tool for Data Mining and Analysis of Lipidomics Datasets". en. In: *J. Proteome Res.* 19.7 (July 2020), pp. 2890–2897.

[11] Martijn R Molenaar et al. "LION/web: a web-based ontology enrichment tool for lipidomic data analysis". en. In: *Gigascience* 8.6 (June 2019).

[12] Caroline Gaud et al. "BioPAN: a web-based tool to explore mammalian lipidome metabolic pathways on LIPID MAPS". en. In: *F1000Res.* 10 (Jan. 2021), p. 4.

[13] Mariia Emelianova et al. "Shiny GATOM: omics-based identification of regulated metabolic modules in atom transition networks". In: *Nucleic Acids Research* 50.W1 (May 2022), W690–W696.
